# More ornamented females produce higher-quality offspring in a socially monogamous bird: an experimental study in the great tit (*Parus major*)

**DOI:** 10.1186/1742-9994-10-14

**Published:** 2013-03-25

**Authors:** Vladimír Remeš, Beata Matysioková

**Affiliations:** 1 Department of Zoology & Laboratory of Ornithology, Faculty of Science, Palacky University, 17. listopadu 50, 77146, Olomouc, Czech Republic

**Keywords:** Carotenoids, Coloration, Great tit, Immunity, Melanins, Mutual choice, Ornaments, Parental care

## Abstract

**Introduction:**

Animals are often conspicuously colored and explanations range from aposematism and mimicry to sexual selection. Although sexual selection explains vivid coloration in males, functional significance of vivid coloration in females of socially monogamous species remains unclear. The hypothesis of mutual mate choice predicts that more ornamented females produce offspring of higher quality. We tested this prediction in the great tit (*Parus major*), a small, insectivorous, socially monogamous passerine.

**Results:**

In both females and males we quantified three ornaments that have been hypothesized to have signaling role in this species (size of black breast stripe, carotenoid chroma of yellow breast feathers, immaculateness of the white cheek). We swapped broods between nests soon after hatching, thus separating genetic plus pre-hatching vs. post-hatching effects on offspring performance. Body mass of offspring at 14 days of age was positively related to the area of black breast stripe of genetic mothers. Immune response to a novel antigen (phytohaemagglutinin) at 14 days of age was positively related to the immaculateness of the white cheek patch of both genetic and foster mothers.

**Conclusions:**

We showed that females with more elaborate ornaments produced higher-quality offspring and we discuss potential proximate mechanisms of these relationships. We conclude that as more elaborate ornaments were reliable signals of offspring quality, direct selection by male mate choice might have been responsible for the evolution and/or maintenance of these signaling traits in females.

## Introduction

Ecological and behavioral functions of animal coloration have traditionally attracted much attention [1,2]. In case of colors and color patterns matching the surroundings, the straightforward interpretation is an adaptation to avoid attack [3]. However, in case of vivid, visible colors the problem is why selection should favor a conspicuous phenotype potentially attracting the attention of enemies. Besides aposematism and mimicry, the leading hypothesis for the evolution of conspicuous color patterns has been sexual selection [4].

Success in sexual selection is given by the ability of an individual to persuade a member of the opposite sex into mating. Conspicuous coloration and ornamentation is at its highest in males of socially polygynous species, especially birds, where the intensity of sexual selection is high [5]. This has a good evolutionary rationale: choosy females pick the most ornamented males for mating, because these males provide superior resources or genes for their offspring and they compete for access to females [6,7]; but see [8]. This logic has been successfully applied and tested on sex role reversed species, where females are aggressive, territorial and conspicuous while males provide most of parental care and are less colorful [4,9]. In these instances, strong sexual selection apparently led to the evolution of conspicuous female coloration. However, most avian species are socially monogamous where both pair members care for the brood [10], but females in many of these species are still as conspicuously colored as males. Why is it so?

Recent research has paid attention to functional significance of female coloration in socially monogamous species [11]. At least three hypotheses were advanced. First, female coloration might be driven by selection on male coloration through intersexual genetic correlation [12]. Second, intrasexual competition for resources [13,14] can lead to the evolution of ornaments signaling competitive ability [9]. Third, mutual choice of partners can lead even in socially monogamous species to the evolution of ornaments signaling individual quality in terms of good genes or parenting abilities [15,16]. This last hypothesis assumes that more ornamented females are of higher heritable quality (survival, resistance to parasites etc.) and/or provide better parental care in terms of positive effects on offspring quality and survival.

**Figure 1:**
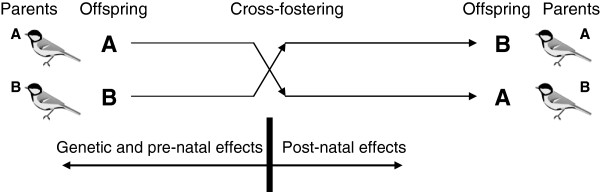
**Design of the cross**-**fostering experiment.** Offspring from two nests (nests **A** and **B**) were swapped two days after hatching. They were raised thereafter by foster parents (parents **A** raised offspring **B** and vice versa). We expected that if the performance of the young was predicted by ornaments of the genetic mother, it would be indicative of genetic and/or pre-natal maternal effects. If the performance was linked to ornaments of the foster mother, it would be indicative of post-natal effects. The picture of the great tit by L. Shyamal was used under the Creative Commons Licence 2.5 (http://creativecommons.org/licenses/by/2.5/deed.en).

In contrast to numerous studies of male mate choice in relation to ornaments (e.g. [17]–[19]), only a handful of studies looked at the relationship between female ornamentation and offspring quality in fish [20,21], lizards [22] or socially monogamous birds [23]–[28]. Moreover, few of them studied multiple female ornaments based on different proximate coloring mechanisms [28,29]. Here, we set forth to investigate this topic using a cross-fostering experiment coupled with brood size manipulation on a large number of nests across two breeding seasons. We chose the great tit (*Parus major*) as our model, because it is a socially monogamous species with female coloration very similar to that of males [30] and bearing multiple colorful patches based on carotenoids, melanins and structure-based mechanisms [31,32]. We exchanged whole broods between pairs of nests (Figure 1), quantified three color patches in females and asked whether the degree of female ornamentation predicted offspring survival and quality (body mass and size, immune responsiveness). We expected that if the mutual choice hypothesis was correct, ornaments of genetic and/or foster mothers would be positively associated with the quality of offspring; moreover, this relationship could be stronger under less favorable conditions (enlarged broods, [33]). This would not be so under the other two hypotheses (i.e., genetic correlation and social competition) and thus our experimental design provided a strong inference in testing competing hypotheses for the evolution of female conspicuous coloration.

## Results

### Female ornaments and offspring performance

**Figure 2:**
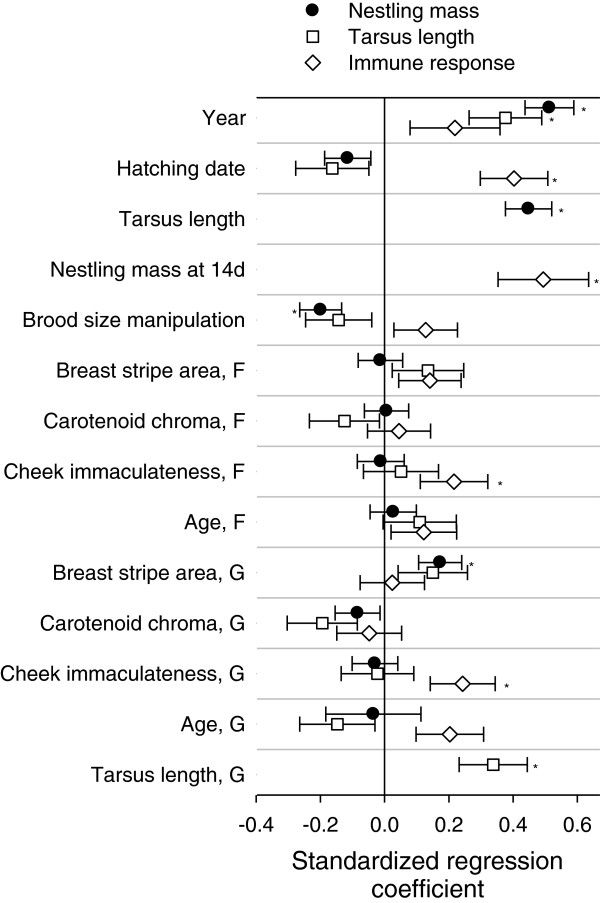
**Standardized regression coefficients (1 SE) from linear regression models.** Response variables were nestling mass, tarsus length, and wing web swelling response (an index of immune response) at 14d of age. F – foster mother, G – genetic mother. Asterisks denote statistically significant factors.

**Figure 3:**
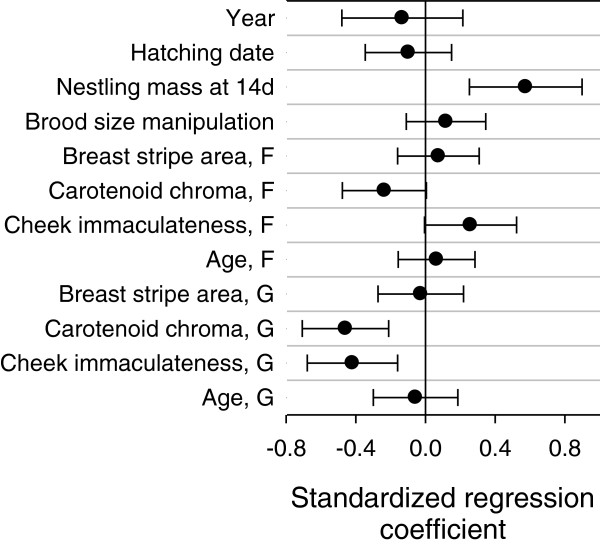
**Standardized regression coefficients (1 SE) on a logit scale from a generalized linear regression model.** The response variable was survival of nestlings until fledging. F – foster mother, G – genetic mother.

Body mass of offspring at 14d of age was positively related to the area of black breast stripe of genetic mothers; additionally, there were strong effects of tarsus length, year, and brood size manipulation (Table 1, Figure 2). The relationship between nestling body mass and breast stripe of genetic mothers was not confounded by female size, because the correlation between female size (tarsus length) and breast stripe area was low (*r* = 0.17, *P* = 0.13) and the analysis was controlled for offspring tarsus length (see Table 1). Tarsus length was related only to the length of tarsus of the genetic mother and differed between years (Table 1, Figure 2). Immune response to a novel antigen (phytohaemagglutinin, PHA) was positively related to the immaculateness of white cheek patch of both genetic and foster mothers; additionally, there were strong positive effects of nestling body mass and date of hatching (Table 1, Figure 2). Nestling survival was not related to any of the predictors used (Table 1, Figure 3). Parameter estimates from these models are detailed in Additional file 1: Appendix 1.

**Table 1:** Summary of statistical models

	**Body mass**		**Tarsus length**		**Immune response**		**Nestling survival**	
**Factor**	**F**	**P**	**F**	**P**	**F**	**P**	**χ**^**2**^	**P**
Year	**45**.**4**	<**0**.**001**	**11**.**1**	**0**.**001**	2.4	0.124	0.2	0.701
Hatching date	2.6	0.113	2.1	0.157	**14**.**8**	<**0**.**001**	0.2	0.689
Tarsus length	**38**.**5**	<**0**.**001**	----	----	----	----	----	----
Fledging mass	----	----	----	----	**12**.**3**	**0**.**001**	3.3	0.068
Brood size manipulation	**9**.**3**	**0**.**003**	1.9	0.170	1.7	0.201	0.3	0.604
Breast stripe area, F	<0.1	0.847	1.5	0.227	2.1	0.149	0.1	0.753
Carotenoid chroma, F	<0.1	0.929	1.3	0.258	0.2	0.649	1.0	0.321
Cheek immaculateness, F	<0.1	0.873	0.2	0.666	**4**.**3**	**0**.**043**	1.0	0.327
Age, F	0.1	0.710	0.9	0.339	1.4	0.236	0.1	0.770
Breast stripe area, G	**6**.**6**	**0**.**012**	1.9	0.170	0.1	0.813	<0.1	0.913
Carotenoid chroma, G	1.4	0.235	3.2	0.080	0.2	0.633	3.6	0.057
Cheek immaculateness, G	0.2	0.677	<0.1	0.844	**5**.**8**	**0**.**019**	2.8	0.096
Age, G	0.2	0.634	1.6	0.211	3.7	0.058	0.1	0.814
Tarsus length, G	----	----	**10**.**3**	**0**.**002**	----	----	----	----

Results of linear models (body mass, tarsus length, immune responsiveness, DF = 11, 66) and the generalized linear model (nestling survival, DF = 11, 64) analyzing effects of year, season, nestling morphology, brood size manipulation, and foster (F) and genetic (G) mothers' traits on offspring performance. Statistically significant factors are highlighted in bold. ---- means that a particular factor was not included in the model.

By fitting interactions between female breast stripe area and brood size manipulation, we tested for potential differential effect of melanin-based ornaments on offspring performance in broods with good vs. poor rearing environment. None of these interactions was statistically significant (Additional file 1: Appendix 1), suggesting that relationships between offspring quality and female melanin-based ornaments did not differ across rearing environments of different quality.

### Assortative pairing

Out of the three feather ornaments measured, only breast stripe area was larger in males than in females (Additional file 1: Appendix 2). Within sexes, ornaments were correlated weakly with the highest correlation between carotenoid chroma and breast stripe area in males (*r* = 0.29; Additional file 1: Appendix 2). Between sexes, all the correlations between ornaments were less than 0.2 and were not statistically significant. The only exception was a positive correlation between male and female cheek immaculateness (*r* = 0.37; Additional file 1: Appendix 2). As female cheek immaculateness was a significant predictor of offspring immune response (Table 1), we wanted to make sure that this relationship was not driven by male cheek immaculateness (given the positive assortative pairing pattern). We re-run the model for offspring immune response with cheek immaculateness of both the genetic and foster father added. Although this model resulted in very small sample size (n = 42 observations) and thus the effects of female cheek immaculateness were no longer statistically significant, absolute values of standardized regression coefficients did not change much (genetic mother: 0.21, SE = 0.16; foster mother: 0.24, SE = 0.17; compare with Additional file 1: Appendix 1) and were higher than coefficients for male cheek immaculateness (putative genetic father: 0.04, SE = 0.14; foster father: 0.15, SE = 0.15).

### Feeding rate

Female feeding rate (mean = 17.2 feeds per hour, SD = 12.15) did not correlate with male feeding rate (mean = 21.5 feeds per hour, SD = 10.35; *r* = 0.16, *P* = 0.167, n = 79). Out of the covariates tested, female feeding rate increased with the number of nestlings and was higher in 2007 than 2006. Other covariates (time of day, ambient temperature, day of season) were not significant and were thus excluded. Male feeding rate increased only with the number of nestlings; other covariates were not significant and were excluded. Full statistical results of these models are available in Additional file 1: Appendix 3.

We then added female age and ornaments to the above-selected models. Female feeding rate correlated neither with any of the three female ornaments nor female age. Similarly, male feeding rate correlated neither with any of the three female ornaments nor female age indicating that there was no differential allocation. Full statistical results of these models are available in Additional file 1: Appendix 3.

## Discussion

We found that females with more elaborate melanin-based ornaments (size of the black breast stripe) and ornaments produced by a combination of melanin coloration and white feathers (immaculateness of white cheek patch) produced heavier young with stronger immune response to a novel antigen. These effects were not confounded by the quality of males. Moreover, there was no evidence for differential allocation by males during nestling feeding (this study) or incubation (no relationship between male incubation feeding and female ornaments, unpublished results; see also [34]) in relation to any of the female ornaments considered. Consequently, males might benefit from choosing females with large breast stripes and immaculate cheeks as mates.

### Proximate mechanisms

Pre-natal maternal effects or genes might be responsible for the relationship between ornament elaboration in *genetic* mothers and nestling mass and immune response at 14d of age in the offspring raised in a foster nest. In this population of the great tit, females with more immaculate white cheeks allocated more vitamins E and A and β-caroten to egg yolk [35]. As carotenoids are potent immunostimulants [36], this allocation pattern could have driven the observed relationship between cheek immaculateness of genetic mothers and offspring immune response. On the other hand, in our population of the great tit, there was no relationship between female breast stripe size and any of the measured components of the pre-natal maternal care: egg and yolk mass, yolk antioxidant and androgen concentration or amount, or the intensity of incubation attentiveness [34,35,37]. Consequently, alternative mechanisms must be sought for the relationship between breast stripe size of genetic mothers and offspring body mass. First, some other component of pre-natal maternal investment might have been responsible. For example, egg white contains numerous antibacterial proteins that are essential for the defense against infections [38]. Similarly, mothers allocate antibodies into egg yolk, both constitutive and induced, which are often strongly linked to offspring performance [39]. Second, the relationship between offspring mass and female breast stripe size might have been mediated by genes. The expression of melanin-based ornaments is often strongly genetically determined [1]. Moreover, the genetic machinery producing melanin-based ornaments is intricately connected to other bodily systems by a web of pleiotropic genetic relationships [40].

*Foster* mothers with more immaculate white cheeks raised nestlings with stronger immune response to a novel antigen. This relationship could not have been generated by genes and thus we might ask which behavioral mechanism was involved. We showed that feeding rate is a reliable proxy of the biomass brought by parents to the nestlings. However, there was no relationship between feeding rate and any of the ornaments investigated. Thus, food quantity did not generate the observed relationship. However, there are other important components of post-natal care that were not quantified in our study. First, although we studied food quantity, more ornamented females might have supplied food of higher quality (e.g. with more nutrients or antioxidants), which could positively affected the nestlings they cared for. Second, brooding by the mother is critical for thermoregulation of ectothermic nestlings and it enables the young to save energy that might be consequently allocated to other energy-demanding physiological systems, e.g. the immune response. Nest sanitation is critical for keeping the nest free of blood-sucking parasites [41] that can decrease condition and performance of nestlings [42,43], especially in species with high loads of parasites [44]. Consequently, higher investment of more ornamented females to nest sanitation could have led to higher performance of nestlings in terms of immune response to a novel antigen.

### Different types of ornaments

Signaling contents of ornaments might differ based on the proximate mechanism generating feather colors. Traditionally, carotenoid-based ornaments were considered condition-dependent due to their plasticity in relation to environment (e.g. food, parasites). On the contrary, melanin-based ornaments were viewed as genetically determined with low potential for signaling condition or individual quality [1,2]. However, it has been recently demonstrated that the difference is not so clear-cut and that melanin-based ornaments might also be plastic in relation to environmental conditions: a metaanalysis of experimental studies showed that the correlation between condition and ornament expression is similar for carotenoid- and melanin-based ornaments [45]. Thus, the potential of female melanin-based ornaments to convey information on individual condition and quality is not less than in carotenoid-based ornaments. In line with this view, barn owl (*Tyto alba*) genetic mothers with larger black, melanin-based spots produced offspring with stronger antibody response [23] and higher resistance to parasites [24], even though these were raised in foster nests. Similarly, alpine swift (*Apus melba*) offspring of darker fathers (color based on eumelanins) raised in foster nests grew their wings more rapidly in experimentally enlarged broods, a difference that was not detected in reduced broods [33].

We found the immaculateness of white cheek in female great tits to be a good predictor of offspring performance. It might be no coincidence that a major component of this signal is based on melanin-colored feathers, because the other melanin-based ornament we confirmed here as a predictor of offspring mass, the black breast stripe, seems to be an important signaling trait across different populations of the great tit. In males, size of the breast stripe signals offspring viability [46], nest guarding [47] and nest defense potential [48]. It also signals competitive abilities in birds foraging in winter flocks [49]. On the contrary, signaling content of yellow breast feathers (see [50]) seems to be unclear given mixed results of previous studies [34,35,37,51]–[54]. It is also interesting to note that the area of the black breast stripe is sexually dimorphic in the great tit (Additional file 1: Appendix 2). Thus, even ornaments that are more elaborated in males as compared to females can have important signaling functions in females.

### Offspring traits

To be biologically relevant, traits we measured on offspring while in the nest must be a reliable proxy of the overall performance of young once they leave the nest. Traits that proved to be related to the elaboration of female ornaments in this study were body mass and immune response at 14d of age. Offspring body mass shortly before or at fledging was demonstrated to be a good indicator of post-fledging survival in numerous species of birds [55]. More importantly, offspring pre-fledging mass was showed to predict juvenile survival and adult mass in several populations of the great tit [56]–[60]. Immune response to PHA was considered to be an index of cell-mediated immune responsiveness. This interpretation was sometimes suggested to be overly simplistic [61]. However, the intensity of the response is species-specific [62] and thus certainly reflects important biological processes. Moreover, there is often a positive correlation between the intensity of this response and both adult and post-fledging survival in birds [63]–[65]; metaanalysis in [66]. Thus, both body mass and PHA-based immune responses have a good potential to indicate offspring quality and performance. In such a case, female melanin-based ornaments would signal future offspring quality and it would pay males to choose females with large breast stripes and immaculate cheeks as mates.

### Positive or negative relationships?

It was suggested that the elaboration of female ornaments is costly and thus more ornamented females may have lower fecundity or offspring with poor survival [9]. For example, offspring of more colorful Arctic charr (*Salvelinus alpinus*) females survived poorly [20] or stickleback (*Gasterosteus aculeatus*) females with more elaborate carotenoid-based coloration of pelvic spines allocated less carotenoids into the eggs [67], but see [68]. In such cases, female ornaments would not be signals of higher-quality offspring. However, it seems that this mechanism can be more important in organisms where the allocation of resources into ornaments vs. reproduction is dynamic, because the resources are allocated into these two functions at the same time (e.g. fish). In birds, feather ornaments are typically grown several months apart from the reproductive season. Thus, potential for the direct trade off between ornaments and reproduction is greatly diminished.

## Conclusions

In sum, we performed an experimental study investigating relationships between multiple female ornaments and offspring performance in a socially monogamous songbird. We found out that the immaculateness of white cheek of both genetic and foster mothers and the size of the melanin-based black breast stripe of genetic mothers predicted performance of offspring on the nest. More ornamented females produced heavier young with stronger immune response to a novel antigen. These two traits are often linked to higher post-fledging survival of the young. Thus, it might pay to the male to choose the female with immaculate cheeks and large breast stripe as a mate. Females seem to signal their parenting quality by the immaculateness of white cheeks. Consequently, sexual selection might have been responsible for the elaboration of these ornaments in females, although it remains to be established whether males pay attention to female ornamentation when choosing the mate in the great tit.

## Materials and methods

### General fieldwork

This work was conducted on three adjacent nest-box plots (188 nest-boxes in total) in a deciduous forest near Grygov (49°31´N, 17°19´E, 205 m a.s.l.) in eastern Czech Republic. The forest is dominated by lime *Tilia* spp. and oak *Quercus* spp. with interspersed ash *Fraxinus excelsior*, hornbeam *Carpinus betulus*, and alder *Alnus glutinosa*. Nest-boxes are placed about 1.6 m above ground and besides great tits are inhabited by collared flycatchers *Ficedula albicollis*, blue tits *Cyanistes caeruleus*, and nuthatches *Sitta europea*. Their design is described in [69].

Fieldwork was carried out in 2006 and 2007 from early April until mid-June. We checked nest-boxes daily to record laying of the first egg and final clutch size. Thereafter, we checked nest-boxes daily around the expected day of hatching to record hatching day. The day when the first young hatched is day 0. During feeding of nestlings (median age of young for females = 7 days, for males = 9 days, range in both cases 6 – 11 days), we captured parents in the nest-box. We captured females at all the nests except one (n = 85). However, because of time constraints, we captured males only at a subset of nests (n = 68). We measured their tarsus length with a digital caliper (nearest 0.01 mm) and weighed them on a spring Pesola balance (nearest 0.125 g). From each bird we took 10 to 15 yellow feathers from the upper right part of breast for later spectrophotometric analysis. We photographed the bird's white cheek (i.e., right side of the head) and breast with a digital camera (Panasonic DMC-FZ5). While taking a picture of the cheek, the bird was held in a standardized position on its left side; while taking a picture of the breast, the bird was held outstretched by its tarsi and beak and photographed together with a ruler from a standard distance (see [52]). All measurements and photographs were taken by VR. We determined the age of birds based on their plumage as one year old or older [70].

We also quantified several components of parental care, both pre-natal and post-natal. Here, we detail only methods and results for parental feeding rates, because results on pre-natal components (egg mass and composition) and incubation behavior were presented elsewhere [34,35,37]. On day 8 (mean = 8.22, range = 8 – 10, n = 86) we videotaped feeding parents from the distance of ca. 5 – 10 m for 90 min. We calculated the number of feeding visits by males and females, which was our estimate of parental feeding rates. To make sure that our feeding rates reflected prey biomass brought to the young, we videotaped parents feeding the young (8 – 12 days old) using miniature cameras installed within the nest boxes (recordings of 90 min, n = 68 nests). In the computer, we took a photo of every prey item, identified it and measured its length. For caterpillars (70% of prey items), which have regular and uniform shape, we also calculated volume of the prey (based on the length and width and assuming cylindrical shape). To make our inference robust, we also assigned prey items into one of three size categories (small, medium, large). Correlation between feeding rate per 90 min and biomass of prey was 0.97 for prey categories, 0.94 for prey length, and 0.70 for caterpillar volume. These high correlations stem from the fact that great tit parents bring always only one prey item and confirm that feeding rate is a good proxy for prey biomass brought to the young on the nest.

On day 13, we measured the thickness of left wing web with a constant-pressure gauge (Mitutoyo PK-1012E). We took the measurement twice and averaged them. We sterilized the wing web with ethanol and injected 0.1 mg of phytohaemagglutinin (PHA) diluted in 20 μl of phosphate buffer (PHA-P, L8754, Sigma-Aldrich) with a disposable syringe (0.3x12 mm). The whole procedure took for every nest always less than 25 minutes. Twenty-four hours later (max. ± 20 min) we re-measured the swollen wing web in the same way as previously. We calculated the strength of reaction to PHA as the difference in thickness between the two measurements spanning 24 hours. On day 14, we also measured tarsus length of each young with a digital caliper (nearest 0.01 mm), weighed it on an electronic balance (nearest 0.1 g), and measured its wing length with a ruler (nearest 0.5 mm). We followed all nests until fledging to record the number of young that successfully fledged.

### Cross-fostering and brood size manipulation

Two days after the first young in the clutch hatched, we performed a cross-fostering experiment by swapping whole clutches between pairs of nests – dyads (Figure 1). We assigned nests to dyads based on their same hatching day. We created both control nests with unchanged brood size (by exchanging whole broods between nests with the same brood size) and nests with experimentally enlarged or reduced brood size (by exchanging whole broods between nests differing in brood size; difference of 1 – 4 nestlings). There was no difference in brood size in 22 nests, whereas broods differed by at least one nestling in 64 nests (by 1 nestling in 26 cases, 2 nestlings in 18 cases, 3 nestlings in 12 cases, and 4 nestlings in 8 cases). The process of brood size manipulation took on average 9.8 minutes per nest (SD = 3.9, range = 3.5 to 20.0 min, n = 86 nests). Brood size treatments were allocated randomly with respect to plumage traits, as there was no relationship between brood size manipulation (brood size difference from −4 through 0 to +4 chicks, see above) and breast stripe size (linear regression; females: F_1,83_ = 0.1, *P* = 0.826, males: F_1,66_ = 0.2, *P* = 0.625), carotenoid chroma (females: F_1,81_ < 0.1, *P* = 0.966, males: F_1,64_ = 2.4, *P* = 0.129) or cheek immaculateness (females: F_1,83_ < 0.1, *P* = 0.952, males: F_1,62_ = 0.1, *P* = 0.799). Birds were handled based on the ringing permission to V. Remeš (no. 1051, Czech Bird Ringing Centre). The study complies with the current laws of the Czech Republic and was approved by the Ethical Committee of Palacky University (without reference number).

### Feather coloration

We chose to analyze the following characteristics of feather coloration: area of the black breast stripe [47], carotenoid chroma of yellow breast feathers [71], and immaculateness of the white cheek [72]. We analyzed photos of breast and cheek in Adobe Photoshop CS3 Extended. We used quick selection tool to roughly delimit the black stripe or the white cheek. Then we manually finished the selection so that it was as precise as possible and measured the surface area of the stripe or cheek. We used a ruler photographed with every bird to adjust the scale of each photo and to obtain absolute surface area (in cm^2^) and in the case of the cheek also perimeter (in cm). We defined stripe surface as the area of the black feathers between the point of inflexion, where the ventral stripe widens to a throat patch, and the posterior end of the stripe [52]. We calculated immaculateness of the white cheek as 4*π*(area/perimeter^2^), which served as an index to measure regularity of the cheek's borders. It is equivalent to the index used by [72] and the value of 1 indicates perfect circle, whereas lower values (approaching zero) indicate shapes with lower area for a given perimeter. All measurements were taken by BM. To assess repeatability, a different observer measured a subsample of photos. Repeatability, calculated as the intraclass correlation coefficient [73], was high for both stripe area (*r*_i_ = 0.87, *P* < 0.001, n = 75) and cheek immaculateness (*r*_i_ = 0.89, *P* < 0.001, n = 75).

We quantified reflectance spectra of yellow feathers sampled from the breast using standard procedures [74]. We used 10–15 feathers from each bird, which is enough to obtain reliable values from our study species [75]. We used an Avantes AvaSpec-2048 fiber optic spectrometer together with an AvaLight-XE xenon pulsed light source and WS-2 white reference tile. The probe was used both to provide light and to sample reflected light and was held perpendicular to feather surface. We took five readings from different parts of each set of feathers. Feathers were arranged on a black, nonreflective surface so that the underlying surface was completely covered and not visible.

We obtained reflectance (%) from 320 to 700 nm in 1-nm increments. We calculated carotenoid chroma as R700 minus R450, divided by R700, where R700 is reflectance at 700 nm and R450 reflectance at 450 nm. We use carotenoid chroma here because it reflects the amount of yellow carotenoids (lutein and zeaxanthin) in breast feathers in the great tit [71]. Hue might be a better measure of carotenoid concentration in saturated carotenoid-based colors [74], p. 82). However, our reflectance spectra always had reasonable reflectance at 450 nm, where lutein and zeaxanthin absorb maximally (analyzed on a broader sample, females: mean = 14.2%, range: 9.3 to 22.5%, n = 128; males: mean = 14.7%, range: 7.8 to 24.4%, n = 101). This indicates that our carotenoid-based color was not saturated and that is why we used carotenoid chroma. In statistical analyses we always used the average chroma calculated from the five readings from each set of feathers. To assess repeatability of our measurements, in a subsample of feathers we arranged feathers anew and took another five readings and again averaged the carotenoid chroma calculated from them. We calculated repeatability of these two average estimates of carotenoid chroma as an intraclass correlation coefficient [73], which was sufficiently high (*r*_i_ = 0.85, *P* < 0.001, n = 55).

### Statistical analyses

Our main aim was to model offspring performance as a function of female multiple ornaments. Due to our cross-fostering experiment, we were able to use simultaneously ornaments of the genetic and rearing females as predictors. As further predictors, we used age of both genetic and rearing females (1y old vs. older), year, hatching date, and brood size manipulation (as a continuous variable ranging from −4 through 0 to +4). To keep our models at reasonable size, we did not fit interactions. The only exception were interactions between breast stripe size and brood size manipulation, because it has been demonstrated that melanin-based ornaments can have stronger predictive power under environmentally unfavorable conditions, e.g. in enlarged broods [33]. However, none of these interactions were statistically significant and were thus excluded from the models (results of these tests are reported in Additional file 1: Appendix 1).

As a response variable, we used average values for all nestlings in the nest of the following offspring traits measured at 14d of age: body mass (g), tarsus length (mm), and wing web swelling as an index of immune response (mm). As another response variable, we used survival of nestlings from cross-fostering until fledging. We modeled survival as the binomial ratio with no. of fledged in the numerator (events) and no. of cross-fostered into the respective nest in the denominator (trials). We used logit link function. We conducted these analyses in R language using functions lm and glm.

It is difficult to select important predictor variables when analyzing observational data. When judging importance of individual predictors in the analyses of offspring performance, we used F-tests in full linear models and likelihood ratio χ^2^ tests in full generalized linear models. Except in case of interactions (see above), we did not use stepwise procedures, because they might lead to biased results [76]; moreover, when the predictors are not correlated, parameter estimates from full vs. minimum models obtained by stepwise procedures are very close [77]. In addition to p-values we focused on standardized regression coefficients as a measure of effect size.

No male was sampled in more than one season. Sixteen females were sampled in both seasons, and 54 females in one season only. For the 16 females sampled in both years, we calculated repeatability of offspring quality defined as intraclass correlation coefficient [73]. We used Proc Varcomp of SAS and calculated repeatability as: variance component of female / (variance component of female + error variance component). We calculated repeatabilities for both genetic and rearing females. All estimated repeatabilities were zero, except for the genetic female effect on offspring tarsus length at 14d of age, which was 0.15. Because of the absence of data clustering by females, we did not used mixed models. However, to make sure that our analyses were robust, we also repeated the analyses with female as a random factor. We obtained qualitatively identical results (results not presented).

Variance inflation factors in all the models were less than 1.6 for all predictors except four, where they were less than 3.0. This indicated that there were no problems with collinearity. We checked the models to conform to the requirements of homoscedasticity, normal distribution and linearity of residuals. Female feeding rate and offspring immune response were square-root transformed. All tests were two-tailed. Sample sizes slightly differ in individual analyses because of missing data points for certain variables.

## Competing interests

The authors declare that they have no competing interests.

## Authors’ contributions

VR conceived the study, analyzed the data, and wrote the first draft of the MS. BM quantified all ornaments and contributed to writing and revising the MS. Both authors collected the data in the field. Both authors read and approved the final manuscript.

## Supplementary Material

Additional file 1 **Appendix 1.** Detailed results of statistical modeling of offspring traits. **Appendix 2.** Descriptive characteristics of, and correlations between, feather ornaments in the Great Tit. **Appendix 3.** Detailed results of the analyses of feeding rates. Click here for file
