# Supplementary material for: More ornamented females produce higher-quality offspring in a socially monogamous bird: an experimental study in the great tit (Parus major)
Source: Front Zool. 2013 Mar 25;10:14. doi: 10.1186/1742-9994-10-14 (PMC3617070; doi:10.1186/1742-9994-10-14)
Supplement: Additional file 1 — Appendix 1. Detailed results of statistical modeling of offspring traits. Appendix 2. Descriptive characteristics of, and correlations between, feather ornaments in the Great Tit. Appendix 3. Detailed results of the analyses of feeding rates. [file 1742-9994-10-14-S1.doc]

**Appendices**

**Appendix 1 Detailed results of statistical modeling of offspring traits.**

Table A1.1 Parameter estimates from linear models (body mass, tarsus length, immune responsiveness) and the generalized linear model (nestling survival; logit scale) analyzing effects of year, season, nestling morphology, brood size manipulation, and foster (F) and genetic (G) mothers' traits on offspring performance. All variables were standardized. Statistically significant factors are highlighted in bold. The same estimates are presented in Figures 2 and 3. ---- means that a particular factor was not included in the model.

|  | Body mass | | Tarsus length | | Immune responsiveness | | Nestling survival | |
| --- | --- | --- | --- | --- | --- | --- | --- | --- |
| FACTOR | Estimate | SE | Estimate | SE | Estimate | SE | Estimate | SE |
| Year | **0.513** | **0.076** | **0.376** | **0.113** | 0.219 | 0.140 | 0.133 | 0.347 |
| Hatching date | −0.115 | 0.072 | −0.163 | 0.114 | **0.403** | **0.105** | −0.098 | 0.248 |
| Tarsus length | **0.448** | **0.072** | ---- | ---- | ---- | ---- | ---- | ---- |
| Fledging mass | ---- | ---- | ---- | ---- | **0.494** | **0.141** | 0.575 | 0.323 |
| Brood size manipulation | **−0.199** | **0.065** | −0.143 | 0.103 | 0.128 | 0.099 | 0.118 | 0.228 |
| Breast stripe area, F | −0.013 | 0.069 | 0.135 | 0.111 | 0.141 | 0.097 | 0.074 | 0.234 |
| Carotenoid chroma, F | 0.006 | 0.069 | −0.125 | 0.109 | 0.045 | 0.098 | −0.236 | 0.241 |
| Cheek immaculateness, F | −0.012 | 0.073 | 0.051 | 0.117 | **0.216** | **0.105** | 0.258 | 0.264 |
| Age, F | 0.027 | 0.072 | 0.109 | 0.114 | 0.122 | 0.102 | 0.064 | 0.220 |
| Breast stripe area, G | **0.173** | **0.067** | 0.150 | 0.108 | 0.024 | 0.100 | −0.027 | 0.245 |
| Carotenoid chroma, G | −0.084 | 0.070 | −0.194 | 0.109 | −0.048 | 0.101 | −0.459 | 0.248 |
| Cheek immaculateness, G | −0.030 | 0.071 | −0.022 | 0.113 | **0.243** | **0.101** | −0.419 | 0.259 |
| Age, G | −0.035 | 0.148 | −0.147 | 0.117 | 0.203 | 0.105 | 0.057 | 0.242 |
| Tarsus length, G | ---- | ---- | **0.338** | **0.106** | ---- | ---- | ---- | ---- |

Table A1.2 Results of testing for interaction between breast stripe area in both foster (F) and genetic (G) mothers and brood size manipulation. As these interactions were not statistically significant, they were excluded from the models (see Methods for justification and further details).

|  | Body mass | | Tarsus length | | Immune response | | Nestling survival | |
| --- | --- | --- | --- | --- | --- | --- | --- | --- |
| INTERACTION of brood size manipulation with: | F | P | F | P | F | P | χ^2^ | P |
| Breast stripe area, F | 0.9 | 0.357 | 0.1 | 0.825 | 0.6 | 0.453 | 0.7 | 0.410 |
| Breast stripe area, G | 1.7 | 0.194 | 0.2 | 0.697 | <0.1 | 0.936 | 0.1 | 0.759 |

**Appendix 2 Descriptive characteristics of, and correlations between, feather ornaments in the Great Tit.**

Table A2.1 Descriptive characteristics of female and male ornaments.

|  | Mean | SD | N |
| --- | --- | --- | --- |
| Cheek immaculateness, F | 0.46 | 0.068 | 85 |
| Cheek immaculateness, M | 0.49 | 0.080 | 64 |
| Carotenoid chroma, F | 0.62 | 0.044 | 83 |
| Carotenoid chroma, M | 0.63 | 0.053 | 66 |
| Breast stripe area, F | 2.37 | 0.854 | 85 |
| Breast stripe area, M | 6.88 | 1.289 | 68 |

Table A2.2 Correlations of ornaments within and between the sexes. Pearson correlation coefficients above the diagonal, sample sizes below. Correlations between the sexes are framed by a black rectangle. Statistically significant correlations are highlighted in bold.

|  | 1 | 2 | 3 | 4 | 5 | 6 |
| --- | --- | --- | --- | --- | --- | --- |
| 1. Cheek immaculateness, F |  | 0.11 | 0.09 | **0.37** | 0.11 | 0.03 |
| 2. Carotenoid chroma, F | 81 |  | 0.12 | 0.18 | −0.16 | 0.13 |
| 3. Breast stripe area, F | 83 | 81 |  | 0.08 | 0.2 | 0.13 |
| 4. Cheek immaculateness, M | 61 | 59 | 61 |  | 0.11 | 0.05 |
| 5. Carotenoid chroma, M | 63 | 63 | 63 | 60 |  | **0.29** |
| 6. Breast stripe area, M | 65 | 63 | 65 | 61 | 63 |  |

**Appendix 3 Detailed results of the analyses of feeding rates.** Female feeding rate was square-root transformed, time of day was log_10_ transformed, and date was centered between years to account for differing timing of the two breeding seasons. Statistically significant factors are highlighted in bold. ---- means that a particular factor was not included in the model.

Table A3.1 Female and male feeding rates in relation to potential covariates.

|  | Female feeding rate | | | Male feeding rate | | |
| --- | --- | --- | --- | --- | --- | --- |
|  | Estimate (SE) | F_1,73_ | P | Estimate (SE) | F_1,73_ | P |
| No of young | **0.30 (0.09)** | **10.9** | **0.002** | **2.47 (0.62)** | **16.1** | **<0.001** |
| Date | −0.05 (0.09) | 0.3 | 0.564 | 0.23 (0.60) | 0.2 | 0.697 |
| Year [2007] | **1.10 (0.36)** | **9.1** | **0.004** | 2.77 (2.47) | 1.3 | 0.265 |
| Time of day | −0.24 (1.23) | <0.1 | 0.846 | 1.25 (8.32) | <0.1 | 0.881 |
| Ambient temperature | 0.02 (0.06) | 0.2 | 0.700 | −0.20 (0.42) | 0.2 | 0.641 |

Table A3.2 Female and male feeding rates in relation to female plumage ornaments and age. Factors statistically significant in Table A3.1 were retained in the models.

|  | Female feeding rate | | | Male feeding rate | | |
| --- | --- | --- | --- | --- | --- | --- |
|  | Estimate (SE) | F_1,69_ | P | Estimate (SE) | F_1,70_ | P |
| No of young | **0.29 (0.09)** | **10.2** | **0.002** | **2.59 (0.59)** | **19.4** | **<0.001** |
| Year [2007] | **1.03 (0.35)** | **8.8** | **0.004** | ---- | ---- | ---- |
| Breast stripe area | −0.23 (0.18) | 1.5 | 0.22 | 1.49 (1.19) | 1.6 | 0.217 |
| Carotenoid chroma | 0.29 (3.47) | <0.1 | 0.933 | −30.29 (22.66) | 1.8 | 0.186 |
| Cheek immaculateness | −1.33 (2.63) | 0.3 | 0.616 | 26.59 (15.58) | 2.9 | 0.092 |
| Age [older than 1y] | −0.28 (0.32) | 0.7 | 0.391 | 2.69 (2.09) | 1.7 | 0.203 |

To make sure that females did not cope differentially with brood size manipulation based on the area of malenin-based breast stripe, we also added “manipulation” as a factor into the model of female feeding rate and fitted an interaction “manipulation*breast stripe area”. This interaction was not statistically significant (F_1,65_ = 0.9, P = 0.406) suggesting that females with differing area of breast stripe fed nestlings at similar rate regardless of brood size manipulation.
